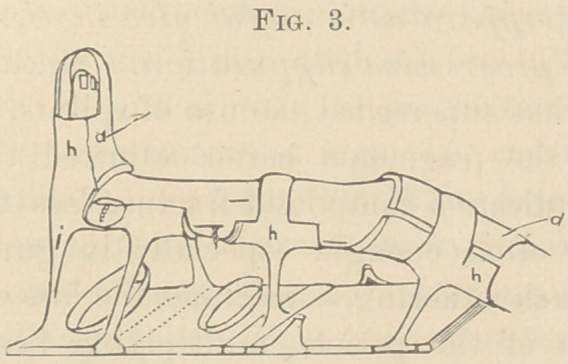# A Simple Means of Securing the Best Posture for the Leg in Case of Compound Fracture

**Published:** 1877-06

**Authors:** H. Banga

**Affiliations:** Chicago


### EXAMINER.

Vol. XXXV.—JUNE, 1877.—No. 6.

### Original Communications.

### A SIMPLE MEANS OF SECURING THE BEST POSTURE FOR THE LEG IN CASE OF COMPOUND FRACTURE.

By Dr. H. Banga, Chicago.

I do not intend, at this time, to discuss the advantages which the plaster of Paris bandage affords in the treatment of compound fracture of the leg. Such a discussion would be as valueless and absurd as the setting forth of the antipyretic power of quinine and its great efficacy in intermittent fever. Since the ingenious adaptation of the plaster of Paris bandage, made during the Crimean campaign, by the great Russian surgeon, Pirogoff, and its subsequent popularization in later wars, it has been almost generally used by surgeons throughout the world, both in civil and military practice. The ease with which it can be applied and the rapidity with which it becomes solid, when it properly and perfectly encases the limb, are advantages which render it so superior to all other immovable apparatus, (especially to starch and silica,) that, if the surgeon has the choice, he should never hesitate to employ the plaster.

He who understands the process of healing in a compound fracture, doubtless knows that, by encasing the leg and opening the bandage in order to expose the wound, the work is not completed, but another, often very tedious, task remains, namely: placing the limb in the most comfortable position.

In this paper, I desire to place before the reader the advantages of an apparatus I have found most suitable for this purpose, but will first offer some general suggestions regarding the posture of the leg and the methods heretofore used.

What is necessary to secure a good posture for the fractured encased leg? The posture is good,

1st, *When the broken bones are kept permanently in proper apposition.* It may seem useless to lay stress on this point, when the leg is once properly put up in the plaster bandage. But it must be borne in mind, that, when the swelling of the limb subsides after a few days, an empty space is formed between the skin and the bandage, thus allowing motion of the ends of the broken bones. By experimenting upon a dead body, lying level upon a table, with a leg fractured, it may be shown that, if the shoulders, or even the head, are raised slightly, the ends of the broken bone will always be displaced, providing they were properly set before the experiment. This difficulty can best be avoided with a patient, *by giving the leg an elevated position.*

2d, *When the circulation is not obstructed,* which is also best assured by elevating the leg.

3d, *When the surgeon can dress the wound in the most convenient manner.* There should be a free space all around the leg, so that the surgeon can examine and even touch, if he deems it necessary, every part, especially the wound, the toes, and the popliteal region, and he should be able to do so without disturbing the patient by raising the leg, etc. This will also enable him to regulate the after-treatment of the wound, as he judges most proper, viz., by the use of ice-bladders, poultices, prolonged irrigation, or, (best of all,) Lister's antiseptic dressing.

4th, *When the attendants are able to keep clean the bed; to apply the bed-pan, etc., without disturbing the patient's comfort.*

5th, *When the patient finds the dressing comfortable.* There is no doubt that in fulfilling the above requirements, there will be the least suffering for the patient. The beginner should bear in mind, that, ordinarily, the patient himself is the best judge of the posture and bandage. If he is comfortable, they may be considered good, and the reverse, if there is any part of which he complains. The surgeon should not hesitate to carefully examine the bed, to lower or to raise the leg, the shoulders, the head, or to cut open or to remove the whole bandage, however faultless it may appear, if the patient complains of any pressure, due care, of course, being taken to determine whether his complaints are without foundation.

Notwithstanding the apparent simplicity of these suggestions, their execution will be found difficult in very many cases, as is proved by the almost numberless apparatuses which have been devised since Jean Louis Petit published the description of his famous fracture-box, and which have all been used in connection with the plaster of Paris bandage. I pass them by, however, since for my purpose it will suffice to show that even suspension, usually considered most suitable, has so many disadvantages, that, if there is a better method claimed, it can not fail receiving *a priori* the attention of the practitioner.

Suspension is accomplished in various ways: by supporting the encased leg in a linen hammock, or wire-splint, attached to a cradle by india rubber tubings, or by suspending the limb from a kind of gallows crossing over the bed by means of cords and iron hooks, fastened at different points in the bandage. Whatever method be adopted, it requires a somewhat complicated apparatus, which doubtless will be found in the well supplied splint-room of a hospital, but which is rarely obtainable on the first day, and often not at all in the country or in an ambulance. Furthermore, the hammocks allow the crest of the leg only to be inspected, and for the dressing of the wound, its removal becomes necessary, so that, if there is a wound at the calf of the leg with progressive suppuration, both surgeon and patient may sometimes dread the time for dressing.

I do not propose to enumerate all the inconveniences experienced in suspending the encased leg, as some of these are referred to elsewhere, but I will at once describe the method which I claim, from experience, to be most suitable for the cases described.

The plaster of Paris bandage will become dry some two hours after its application, and the wound may then be exposed by means of a valvular opening, cut in the bandage. For our apparatus we merely require,

1st, A well planed board, from eight to ten inches in width, and one-fourth inch in thickness, and of about equal length with the leg from the popliteal region to the heel.

2d, Some wooden blocks, each about four inches long, one inch wide, and of different thickness.

3d, Some small wooden wedges.

4th, Some rollers prepared with plaster of Paris, some plaster in powder, some water, and a small vessel in which to mix the plaster and water to the consistency of cream. All these things being in readiness, the apparatus is constructed in the following manner:

The leg is gently raised on a level, about one foot above the bed, by one or two assistants, while the surgeon places the board lengthwise on the bed beneath the leg. The surgeon then piles up the blocks evenly across the board, at two places; viz., beneath the ankle and under the popliteal region. Two pillars, each from six to eight inches high, are thus formed. The leg should then be lowered to rest on these pillars, and to prevent rolling to the sides, the above-mentioned wedges are used. (Fig. 1.) This done, the position of the limb must be carefully examined, for this is to be its final posture. Therefore, the patient should be asked, as to how he feels. Perhaps he wishes the foot slightly raised, which can be done either by placing another small block beneath the ankle, or by taking out a block from under the knee, or by gently moving either pillar upwards or downwards, as may be required to raise or lower the corresponding part of the leg. The upper portion of the bandage around the thigh, should be especially examined, to ascertain if there be any pressure or constriction, which often happens if the knee is bent too much or too little. If overlooked, such pressure will afterwards cause much trouble, often inducing the patient to crumble away the bandage. It is evident, that after having dressed some cases in the manner here described, the surgeon will soon be able to judge at once of the proper height of the pillars, if only he bears in

FIG. 1.

mind that the best posture requires the foot to be the highest part, while the knee-joint may be a little lower and somewhat flexed. He will accomplish, in a short time, what it has taken so many words to describe.

If the position is satisfactory, both to the surgeon and the patient, it is secured as follows: With a well-wetted plaster roller three turns are applied around the plaster bandage near the ankle, closely above or beneath the lower pillar. (Fig. 1 *a.*) On reaching the crest of the bandage after the third turn, the roller is passed obliquely downwards to the edge of the board, (Fig. 1 from *a* to *c*) and underneath the latter, to the opposite edge, from which it is carried upwards back to the crest of the bandage (at *a*). While the roller is passed underneath the board, this with the leg, must, by an assistant, be raised about one inch from the bed. The roller, thus applied, forms a kind of triangle with its base across the board, while the sides reach obliquely down from the crest of the bandages to the edges of the board. This triangular band must be repeated twice more, so that each of its sides contains three layers of roller bandage, and care must be taken to tighten well the roller while going around. Another well-wetted plaster roller is now wound spirally around the inner lateral portion of the triangular band. Beginning at the edge of the board, the spiral turns gradually ascend, the roller being always tightened, and each turn joining closely the foregoing. Where they reach the leg, the roller is cut out, the end spread on the bandage (at *a* Fig. 1). In the same way, spiral turns are then made around the external lateral portion of the triangle. The object of the spiral roller and of the tight application is to double up the three strips which constitute the sides of the triangular band and to transform these bands into two round, slender columns, which we may call *plaster stilts.* The strength of the stilts will be increased, and their appearance greatly improved by coating and smoothening them with plaster cream. After the stilts near the ankle are completed, a second pair is, in like manner, made up under the knee, corresponding with the upper block-pillar. After a few hours, the stilts will be dry and hard, and then, taking away the blocks, the surgeon will find that the leg is very securely and comfortably supported by the plaster-stilts, on a level from six to eight inches above the board—plaster-bandage, stilts and board forming one whole immovable apparatus. (Fig. 2.)

Fig. 2.

When I first employed the method here described, I made three pair of stilts, also one single one, reaching from the sole of the foot to the lower end of the board. (Fig. 3, *i.*) I also thought it necessary to make the stilts of five longitudinal and two spiral layers of roller bandage, and to coat all these with a large quantity of plaster cream. Of course, I produced a very heavy and clumsy apparatus. Later, I learned, with much astonishment, that even a slender column thus constructed, had all the strength required. Even three stilts, two at the knee-joint, and one extending from the foot to the lower edge of the board, (Fig. 3, *i*), would be sufficiently strong to support a leg, but as the single stilt necessitates much extra labor to fasten it to the board, its construction is more tedious and less expeditious than that of a pair of stilts at the region of the ankles. Therefore, after my experience, I would recommend the plan first given as the best; viz., two pair of stilts. The advantages afforded by the apparatus just described are:

1. *There is no difficulty, even in the country, to get the materials we need, or to use them properly.* I think I hardly need say, that the description given of the blocks, the wedges, and the way of setting them up, is to be taken *cum grano salis;* for, as the hospital-surgeon can, at all times, have in

Fig. 3.

readiness in the splint-room sets of blocks, etc., just as well can the ill-provided country practitioner use whatever he finds at hand, as stones, bricks cut in pieces, for blocks, or rags instead of wedges, articles he can obtain, even in the poorest family, should he be obliged to treat there a case of compound fracture of the leg.

2. *The dressing of the wound is easy for the surgeon and painless for the patient.* As every part of the leg is exposed, the surgeon can touch any spot with finger, probe or forceps, or, by bending his head, he can inspect the lower portion of the limb, or the calf, without moving the patient a hair's breadth. The time required for dressing the wound will be much shortened by saving the task of piling up the cushions, pads and sand-bags, and of adjusting splints and suspending strings, and the surgeon will be enabled to shorten the time of his visits, much to his own and the patient's benefit. He is also more independent of the nurses and the stewards,—an advantage of no less importance in an overcrowded ward, than in the country where you are almost always assisted by inexperienced hands. Furthermore, the apparatus will be found very suitable for the treatment of the flesh wound attending the fracture, because vessels can be placed everywhere under the plaster bandage, to receive the discharge, or water running down from it, or out of the drainage tubes. Ice-bladders or poultices can be applied to the bandage without difficulty, For readers acquainted with Lister's antiseptic dressing, which yields, in the grave cases of compound fracture of the leg, the most brilliant results, I will add that my apparatus permits also the application of the spray and carbolized muslin rollers.

5. *The stilt apparatus is useful also in the treatment of erysipelas and progressive suppuration.* Since the plaster of Paris bandage has superseded the use of splints, for the reason that it keeps the fragments better adjusted, these evils undoubtedly complicate a compound fracture less than formerly, yet they occur often enough, especially in gunshot wounds, resulting in much suffering, sometimes the loss of a limb, and even the death of the patient, multiplying besides the surgeon's already tedious labors. To illustrate the application of my method, I append notes of a typical case:

In 1874, a case of compound fracture of the leg, caused by a heavy wagon passing over it, was admitted to my division at the hospital of Basle, Switzerland. Both bones were broken, the tibia splintered, and a lacerated wound, as large as the palm of the hand, was found about two inches above the ankle joint, on the inner side of the leg. The soft parts were also badly bruised. The fracture was at once incased, and after two hours, placed on stilts, as described; a compress, soaked in an antiseptic solution, was laid on the open wound. During the first three days nothing worthy of note occurred, but on the fourth day, the wound became very painful and swollen, copious suppuration taking place, the patient becoming feverish. The next morning he was very ill; had had a chill during the last night, and the wound was more painful and swollen; more pus was discharging. On the inner side of the thigh I noticed the reddish streaks, which indicate lymphangitis; the inguinal glands were also painful and swollen. As the pus seemed to come from above, I enlarged the opening of the bandage upwards, about two inches, thus exposing the suppuration progressing along the long flexor muscles.

A large abscess was there opened. Immediately after the incision, the patient felt greatly relieved, and soon fell asleep for some hours. However, in the evening, the pain became more severe, and the next morning I discovered a pouch connected with the abscess, already opened: the pouch extending downwards and backwards in the calf. The bandage was cut away in the same direction, and to a sufficient extent to fully expose the region, where the redness, swelling and pain betrayed the deep-seated matter. A large incision was made, and the pus allowed to escape through a drainage tube into a vessel beneath the leg. The next ten days the patient did well, the lymphangitis disappeared, *pus bonum et laudabile* was secreted from the wound, mixed with detritus of the bruised tissues. On the morning of the fifteenth day, however, I found the patient once more feverish; he complained of throbbing and burning in the leg. There was no remarkable change about the wound, and the two abscesses before opened seemed to be cicatrized. But on gently examining the skin with the finger, as far as the leg was exposed, a suspicious reddish tint and a certain tenderness about the upper edge of the opening, induced me to still enlarge it in the same direction. I discovered a large abscess, which could only be wholly laid open by cutting away the inner half of the bandage to the knee-joint. Perhaps some one will ask: "How was it possible to take away the inner half of the bandage from two inches above the ankle to the patella, and also a piece as large as the palm from the back, without destroying the whole apparatus?" "Just by means of those stilts," I answer. According to the directions given above, I had put the incased leg on four stilts, *i. e.*, one pair near the ankle, the other pair just below the popliteal region. When the opening in the bandage was first enlarged, the latter was undisturbed, and so it was when I took a piece from the back. Only when the last large abscess was revealed, the inevitable sacrifice of the inner stilt of the upper pair caused some apprehension as to the safety of the structure. However, I could easily secure the apparatus, as I had done before in several cases, that is, before exposing the abscess, I erected a new stilt above the knee-joint. This stilt was made as previously described, with the only difference, that the roller, after crossing beneath the board, was divided so that the end could be spread over the latter. After some three hours, when the new stilt had become dry, I could cut off the inner, upper stilt without any danger, its task being assumed at the same moment by the new stilt.

Changing the stilts is to be regarded as most advisable, and for this reason I append the notes of another of my cases treated in 1874.

After incision of the right ankle joint, I had put up the leg very comfortably by means of two plaster of Paris splints, an upper or dorsal, and a lower or plantar. They were modeled to the leg one hour before the operation, and coated with paraffin, so as to render them waterproof. Afterwards, I applied the stilts. Rather profuse suppuration followed, and on the fourth day a very serious phlebitis, (venæ saphenae magnae,) and two days afterwards erysipelas migrans, which caused several abscesses, so that, at different intervals, I had to take off the whole upper-splint from the ankle-joint to the patella, and I proceeded in about the same manner with the back, taking away at first a piece as large as three fingers, from a little above the insertion of the tendo Achillis. About ten days later, I discovered an abscess on the upper third of the calf. When I perceived, that, by cutting away the necessary amount, the whole back of the leg would be deprived of all support, I proceeded to secure the endangered piece as follows: With a well wetted plaster roller I made two circular turns in the middle of the leg, corresponding to the middle piece of the back splint to be secured. On the third turn I went around only to the middle of the calf, where I ordered my roller to be held in a loop, formed with another plastered roller by an assistant from the opposite side. After the two ends of the loop were clipped and spread on the circular turns, my own roller appeared fastened to the middle of the calf, whence it proceeded perpendicularly downwards to the board, and, whilst an assistant's forefinger held the bandage to the board, I carried it straight across to the edge of the board, crossed it underneath and returned from the opposite side to the foot of the perpendicular band and around it, securing it by spreading the clipped end of my roller on the board. In just the same way I made two more turns with my roller and lastly dressed the stilt by means of spiral turns and coating with plaster cream, as heretofore described. When the stilt was dry, I removed the piece from the back splint, which I had to take away by means of a small saw, after which I found the remaining middle portion of the back splint very properly supporting the calf, as a kind of *pelotte* on the top of the column. (Fig. 3, p.) In this apparatus the patient was kept over three weeks and during this whole time the wound and all the abscesses were constantly in sight and opened as soon as fluctuation was evident. There was no difficulty in dressing the wounds. In spite of the erysipelas the process of granulation proceeded very well, and after fifteen days there was no more fever. After the lapse of three weeks, nearly all the abscesses were cicatrized, and the consolidation of the bones was sufficiently strong to warrant the changing of the remnants of the first apparatus, on about the 25th day after the operation. I believe one should hardly go further than just described in cutting away and mutilating the bandage, yet I think the readers will agree with me, that it is sometimes very convenient for the practitioner, and no less beneficial to the patient, to defer its renewal even for two or three days by the means mentioned. In the case last reported, I would attribute the prompt healing of the wound chiefly to the absolute rest in which the foot and the leg were kept more than three weeks; during the third week this rest was only possible with the stilt put under the calf.

5. *It is almost needless to say that compound fractures of the leg are not the only cases where the stilt apparatus may be used.* The surgeon will find it very suitable also, in the after-treatment of cases of excision of the knee, elbow and ankle joints.

6. *There is no difficulty when the nurses render proper*

*services* to the patient, as with the bed-pan, cleaning the bed, etc.

The apparatus I have described is a modification of the method described some ten years ago by Dr. Ries, Billroth's assistant at Zürich and described in an interesting pamphlet on the plaster of Paris bandage. Dr. Ries puts the encased leg on two or three small blocks, with an upper concavity, fastening the leg and the blocks on a board by means of either an ordinary or a plastered roller. When I entered as house surgeon the hospital of Basle, Switzerland, I learned from my predecessors the method; a vast number of blocks and boards being at hand in the splint-room. But, as the reader will perceive, Dr. Ries' method is rather clumsy; the blocks move very often; there is not a free space all around the leg, etc.; so that I thought it might prove useful to publish a description of my stilt apparatus, because since I first devised it in 1874, I have proved its advantages in many different cases.

Should it be thought that I have employed too many words in explaining a very trifling affair, my response could be best formulated in the apoththegm of Dieffenbach, the great master of our profession: "that in surgery success almost always depends upon the scrupulous and rather pedantic observance of a host of trifles."

## Figures and Tables

**Fig. 1. f1:**
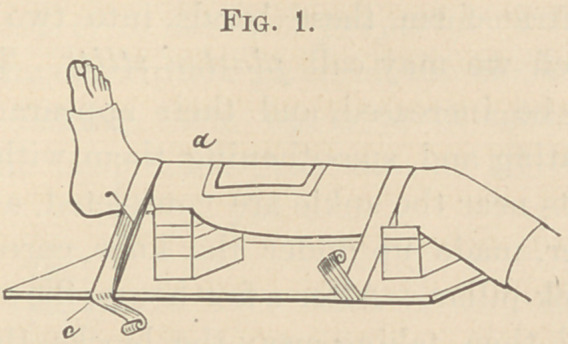


**Fig. 2. f2:**
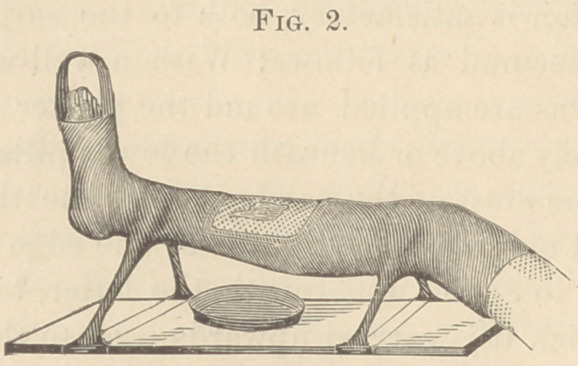


**Fig. 3. f3:**